# PFDN6L Gene Predicts Good Prognosis Associated with Its Inhibition of the Stem-Ness Properties in Hepatocellular Carcinoma

**DOI:** 10.32604/or.2025.067628

**Published:** 2025-11-27

**Authors:** Fangyuan Li, Xiaoyuan Hu, Xiaoge Gao, Ling Liu, Tao Li, Dan He, Jiaxing Cheng, Xiaobiao Ma, Li Li, Chunlei Ge, Hong Yao

**Affiliations:** 1 Cancer Biotherapy Center & Cancer Research Institute, The Third Affiliated Hospital of Kunming Medical University, Yunnan Cancer Hospital, Peking University Cancer Hospital Yunnan, Kunming, 650118, China; 2 Shenzhen Baoan Women’s and Children’s Hospital, Shenzhen, 518100, China; 3 Cancer Institute, Xuzhou Medical University, Xuzhou, 221002, China

**Keywords:** Hepatocellular carcinoma, liver cancer stem cells, prefoldin subunit 6-like protein (PFDN6L), prognosis

## Abstract

**Background:**

Liver cancer stem cells (LCSCs) are recognized as pivotal drivers of hepatocellular carcinoma (HCC) progression; however, the molecular mechanisms maintaining their stem-like phenotype remain largely unresolved. This work investigates the role of prefoldin subunit 6-like protein (PFDN6L) in shaping LCSC traits and promoting or restraining HCC progression.

**Methods:**

PFDN6L, a cytoskeleton-associated chaperone, was studied using multiple *in vitro* assays—cell growth evaluation, cell cycle profiling, and spheroid culture—alongside analyses of stemness-associated markers (SOX2, CD133, CD44). Tumorigenic capacity was assessed in xenograft mouse models, and signaling pathway interrogation was performed to define underlying mechanisms.

**Results:**

In patient samples, higher PFDN6L expression correlated with improved survival outcomes. Forced expression of PFDN6L induced G2/M arrest, diminished sphere formation, and reduced pluripotency marker expression, whereas knockdown accelerated *in vivo* tumor formation. Mechanistic experiments demonstrated that PFDN6L suppresses malignancy by simultaneously dampening AKT and ERK1/2 activation, thereby impairing oncogenic signaling cascades.

**Conclusion:**

PFDN6L acts as a negative regulator of LCSC-driven tumorigenesis. Its dual blockade of AKT and ERK pathways forms the mechanistic basis of its tumor-suppressive action, supporting its potential as a prognostic biomarker and therapeutic target in HCC.

## 1 Introduction

Hepatocellular carcinoma (HCC) is the most prevalent subtype of liver cancer globally, accounting for approximately 75% of hepatic carcinogenesis [1–3]. Numerous risk factors, including hepatitis infection, cirrhosis, alcohol, or aflatoxin B1 (note: it should be aflatoxin B1 rather than just aflatoxin B), among others, are associated with the development of HCC [2]. Over the past 5 years, various drugs such as sorafenib and Lenvatinib (tyrosine kinase inhibitors), immune checkpoint inhibitors, and their combination therapies have significantly enhanced the prognosis of advanced HCC. However, drug resistance and recurrence after surgery still impede their clinical efficacy. With a 5-year survival rate for HCC patients still under 60% [4], identifying new prognostic markers and therapeutic targets is essential to enhance early detection and treatment outcomes. Notably, cancer stem cells (CSCs) represent a distinct tumor cell population characterized by unique functional capabilities. They possess self-renewal capacity, the ability to proliferate indefinitely, and the potential to differentiate into heterogeneous cancer cells [5–7]. The CSCs of HCC, referred to as Liver cancer stem cells (LCSCs), are resistant to conventional anticancer treatments and can evade immune responses [8–10]. They are responsible for treatment resistance, disease recurrence, and metastasis in HCC patients [11]. Consequently, LCSCs are regarded as an effective therapeutic target for HCC to improve the prognosis of patients with this disease [12,13].

The identification of cancer stem cells predominantly depends on tumor surface markers. Currently, CD133 (human prominin-1, PROM1), epithelial cell adhesion molecule (EpCAM), CD90 (Thy-1), CD13 (Aminopeptidase N), CD44, and OV-6 are regarded as the surface markers of LCSCs [14]. Studies have demonstrated that elevated CD133 expression independently predicts survival outcomes and recurrence risk in patients with hepatocellular carcinoma [15]. CD133^+^ HCC cells have been found to exhibit stronger stem cell-like characteristics, such as migration, spheroid formation, and tumorigenicity, via the EGFR-AKT signaling pathway [16]. Recent studies have revealed that the Wnt/β-catenin, Notch, Hedgehog, and TGF-β signaling cascades play critical roles in governing the self-renewal, proliferative capacity, and differentiation of liver cancer stem cells [17,18].

Coiled-coil domain-containing protein 30 (CCDC30) and prefoldin subunit 6-like (PFDN6L) refer to the same gene product. It belongs to the coiled-coil domain protein family. Coiled-coil domains are crucial modules for protein interaction and are frequently involved in multimer formation or binding to other proteins to regulate cytoskeletal dynamics, signal transduction, or molecular chaperone function. Various proteins of the CCDC family have been reported to regulate the biological behaviors of malignant tumors, including cell proliferation, invasion, and metastasis [19]. The PFDN6L gene, a splice variant containing the carboxyl-terminal 572 amino acid residues of CCDC30, was initially identified in the human brain, kidney, and pancreas in 2006 [20]. Although PFDN6L may regulate tumorigenesis or progression, direct experimental evidence is currently lacking. Recent advances in computational frameworks, particularly ensemble methods like the multi-model voting approach for anticancer peptide classification [21], demonstrate significant potential for improving biomarker validation strategies. Such methodologies could enhance prognostic modeling for targets like PFDN6L in future studies.

Our previous proteomics research demonstrated that the inhibition of liver cancer stem cell activity is closely associated with a significant upregulation of the PFDN6L protein level. Given the potential involvement of PFDN6L in modulating liver cancer stem cells, this study focused on elucidating its functional role and the molecular mechanisms governing liver cancer stemness and hepatocellular carcinoma development.

## 2 Materials and Methods

### 2.1 Analysis of Public Databases

To further evaluate the expression profile of PFDN6L in hepatocellular carcinoma (HCC), we analyzed publicly available datasets, including The Cancer Genome Atlas (TCGA) (https://www.cancer.gov/tcga, https://gtexportal.org/). Expression and survival data were retrieved and processed according to the respective database guidelines. To assess the relationship between clinicopathological factors and overall survival in HCC patients, both univariate and multivariate Cox proportional hazards models were utilized. Variables demonstrating a *p*-value below 0.05 in univariate analysis were subsequently incorporated into the multivariate analysis. Corresponding hazard ratios (HRs) with 95% confidence intervals (CIs) were estimated, with statistical significance set at *p* < 0.05.

### 2.2 Cell Culture

The human hepatocellular carcinoma cell lines PLC/PRF/5, HepG2 (sourced from the Shanghai Chinese Academy of Sciences Cell Bank), and Huh7 (established in-house) were maintained in DMEM (Gibco, C11995500BT) supplemented with 10% fetal bovine serum (Gibco, 10099141C), 100 U/mL penicillin, and 100 μg/mL streptomycin (Gibco, 15140122). Prior to use, all cell lines were verified to be free of mycoplasma contamination and authenticated through short tandem repeat (STR) profiling. They were cultured in 37°C and 5% CO_2_ in a humidified atmosphere, in a tissue culture dish (Wuxi Naisi Biotechnology Co., Ltd., WX-CCD-100, Wuxi, China) containing 10 mL of complete culture medium.

### 2.3 Western Blot Analysis

After treatment, PLC/PRF/5, HepG2 and Huh7 cells cultured in cell culture dish were lysed in pre-prepared RIPA buffer (Beyotime, P0013B, Shanghai, China) containing PMSF (Beyotime, ST506, Shanghai, China) on ice. After centrifugation at 13,000× *g* for 15 min, the protein concentration was then detected using BCA protein concentration Kit (Kaiji, KGP902, Nanjing, China). An equal quantity of protein was separated by SDS-PAGE and transferred into PVDF membrane (Millipore, IPVH00010, Burlington, MA, USA), followed by blocking with TBST (APExBIO, K1199, Shanghai, China) incubated with 5% non-fat dried milk for 2 h at ambient temperature. Subsequently, the membrane was incubated overnight with the following primary antibodies at 4°C: PFDN6L antibody 1:1000 dilution (Thermo Fisher, PA5-71297, Waltham, MA, USA), CDC2 antibody 1:4000 dilution (Cell Signaling Technology, #77055 Danvers, MA, USA), P21 antibody 1:3000 dilution(Cell Signaling Technology, #2947, Danvers, MA, USA), P27 anytibody 1:3000 dilution (Cell Signaling Technology, #2552, Danvers, MA, USA), CD133 antibody 1:3000 dilution (Cell Signaling Technology, #5860, Danvers, MA, USA), SOX2 antibody 1:4000 dilution (Cell Signaling Technology, #23064, Danvers, MA, USA), CD44 antibody 1:4000 dilution (Cell Signaling Technology, #37259, Danvers, MA, USA), total-Akt antibody 1:3000 dilution (Cell Signaling Technology, #9272, Danvers, MA, USA), p-Akt antibody 1:5000 dilution(Cell Signaling Technology, #4060, Danvers, MA, USA), total-ERK1/2 antibody 1:3000 dilution (Cell Signaling Technology, #9102, Danvers, MA, USA), p-ERK/2 antibody 1:3000 dilution (Cell Signaling Technology, #4370, Danvers, MA, USA) and GAPDH antibody 1:10000 dilution (Cell Signaling Technology, #2118, Danvers, MA, USA). Subsequently, membranes were incubated at room temperature for 1 h with HRP-conjugated Anti-rabbit IgG (1:10,000; Cell Signaling Technology, #7074) and Anti-mouse IgG (1:10,000; Cell Signaling Technology, #7076). Following washing with TBST (APExBIO, K1199), the chemical signals were finally detected using the Tanon imaging system (Tanon Science & Technology, 5200, Shanghai, China). Quantification of band intensities was performed with ImageJ software (version 1.53t; National Institutes of Health, Bethesda, MD, USA) to detect the gray values of the target protein band and the internal reference protein band. The relative expression intensity of the target protein was calculated by dividing the gray value of the target protein band by that of the internal reference protein band, Statistical analyses were performed utilizing GraphPad Prism software (version 9.5.1; GraphPad Software, LLC, San Diego, CA, USA) to evaluate whether the differences in relative expression intensity among different samples or treatment groups were statistically significant.

### 2.4 Lentiviral Transduction

Lentiviruses encoding PFDN6L (overexpression plasmid) or empty vector (GenePharma, Suzhou, China) were employed to transduce HCC cells (PLC/PRF/5, HepG2, Huh7) cultured in 6-well plates at 60%–70% confluency; cells were infected at an multiplicity of infection (MOI) of 20 with 8 μg/mL polybrene (GenePharma, G0401-1) for 24 h, followed by medium replacement and selection with 2 μg/mL puromycin (GenePharma, G0408-1) for 72 h.

### 2.5 Assessment of Cell Proliferation

Cell proliferation was assessed using the CCK-8 assay (Dojindo, CK04, Kumamoto, Japan). HCC cells (HepG2/Huh7: 1 × 10^3^; PLC/PRF/5: 2 × 10^3^ cells/well) were plated into 96-well plates and cultured for 24, 48, 72, and 96 h. After adding 10 μL of CCK-8 solution, plates were incubated for 2 h at 37°C, and Absorbance at 450 nm was determined using a microplate reader (BioTek Synergy H1, Winooski, VT, USA).

### 2.6 Colony Formation Assay

HCC cells (HepG2/Huh7: 1 × 10^2^; PLC/PRF/5: 1 × 10^3^ cells per well) were plated in 6-well plates and incubated for about 14 days to allow colony formation. Colonies were fixed with methanol (Dingchen Technology Co., LTD, CA14995000, Shijiazhuang, China), stained with 0.1% crystal violet (Saiweier, GC307002, Wuhan, China), and counted under a light microscope (OLYMPUS, CX41-32C02, Tokyo, Japan). All experiments were conducted in triplicate, with data analyzed via Student’s *t*-test.

### 2.7 Cell Cycle Assay

3 ×10^5^ PFDN6L overexpression cells and vector control cells in each well were inoculated into 6-well plates. For synchronization, cells were serum-deprived (0.1% FBS, Gibco, C11995500BT) for 24 h, released into complete medium (10% FBS, Gibco, C11995500BT) for 12 h, harvested by trypsinization (Gibco™, 25200056, USA), washed with Phosphate-Buffered Saline (PBS; Corning, 21-040-CVR, USA), and fixed in ice-cold 70% ethanol (Absolute Ethanol, Merck Millipore, 100983, Darmstadt, Germany) at 4°C overnight. Fixed cells were washed, treated with RNase A (100 μg/mL; RNase A, Thermo Fisher, EN0531, USA), and stained with propidium iodide (PI, 50 μg/mL; Propidium Iodide, Sigma-Aldrich, P4170, St. Louis, MO, USA) for 30 min at 37°C in the dark. Cell cycle profiles were determined using a BD FACSCanto II flow cytometer (BD Biosciences, Model FACSCanto II, V145687, Franklin Lakes, NJ, USA). The gating strategy involved FSC-A vs. SSC-A plots to eliminate debris and identify viable cells, PI-W vs. PI-A plots to isolate singlets, and PI-A histograms applied to singlet populations to evaluate DNA content and determine cell cycle distribution.

### 2.8 Sphere Formation Assay

CD133^+^ PLC/PRF/5 hepatocellular carcinoma cells were magnetically isolated from PFDN6L-overexpressing and control groups using a CD133 MicroBead Kit (Miltenyi Biotec, 130-097-049, Bergisch Gladbach, Germany). Isolated cells were suspended in serum-free tumorsphere medium comprising: The culture medium consisted of DMEM/F12 basal medium (Gibco™, 11330032, USA) supplemented with 20 ng/mL recombinant human EGF (PeproTech, AF-100-15), 20 μg/mL B27 supplement (Gibco™, 17504044, USA), 20 ng/mL recombinant human bFGF (PeproTech, 100-18B, USA), 4 μg/mL recombinant human insulin (Sigma-Aldrich, I9278, USA), and 1% penicillin–streptomycin (Corning, 30-002-CI. USA).Cells were plated at 5 × 10^3^cells/mL in ultra-low attachment 6-well plates (Corning^®^, 3471, USA) with 1.5 mL medium/well and maintained at 37°C/5% CO_2_ for 14 days. Primary tumorspheres were enzymatically dissociated using Accutase (STEMCELL Technologies, 07920, Vancouver, BC, Canada) and re-seeded in 96-well ultra-low attachment plates (Corning^®^, 7007, USA) at densities of 200, 500, and 800 cells/well in 50 μL medium. Fresh pre-warmed medium (50 μL) was supplemented every 72 h. After 14 days, spheres >50 μm in diameter were quantified under an inverted phase-contrast microscope (Leica, DMi8, Leica Microsystems GmbH, Wetzlar, Germany).

### 2.9 Quantitative Reverse Transcription-PCR (qRT-PCR)

Total RNA was extracted from PFDN6L cells and control samples utilizing the ZYMO RESEARCH RNA extraction kit (ZYMO, RNA123, USA). Reverse transcription was performed using the Vazyme reagent kit (RT456, Nanjing, China) strictly following the manufacturer’s instructions. Subsequently, qRT-PCR was performed using the qRT-PCR SYBR^®^ Green Master Mix (Vazyme, qRT-PCR789, China), following the manufacturer’s recommended procedure with precision. Relative gene expression was quantified using the comparative threshold cycle (ΔΔCt) method, with values normalized to the endogenous reference β-actin to ensure result accuracy and reliability. The primers of the PFDN6L and β-actin genes are PFDN6L: Forward, CAGCCTGGAGAAGACCCTTC, Reverse, GGCTCAGGTCCAGGT AGTTG; β-cactin: Forward, CATGTACGTTGCTATCCAGGC, Reverse, CTCCTTAAT GTCACG CACGAT.

### 2.10 Tissue Samples

A total of 104 patients with advanced HCC, treated at the Affiliated Hospital of Xuzhou Medical University between 2009 and 2016, were enrolled in this study. None had undergone chemotherapy or radiotherapy prior to surgery, and relevant clinical data (Table 1) were collected from all participants with informed consent. During surgery, paired tumor and adjacent non-tumorous tissues were obtained; samples for immunohistochemistry (IHC) were immediately fixed in 10% formalin, while in eight cases, portions of both tissue types were large enough to be snap-frozen in liquid nitrogen for subsequent protein analysis. The study protocol was reviewed and approved by the Institutional Medical Ethics Committee of the Affiliated Hospital of Xuzhou Medical University (KYLX2024096) in compliance with the Declaration of Helsinki.

**Table 1:** The relationship between the expression of PFDN6L and clinicopathological features in HCC patients

Variables		PFDN6L (n = 104 cases)		
	n	Low (%)	High (%)	p*
All patients	104	50 (48.07)	54 (51.92)	
Age (years)				0.691
≤60	61	28 (45.90)	33 (54.09)	
>60	43	22 (51.16)	21 (48.84)	
Sex				0.619
Males	85	42 (49.41)	43 (50.58)	
Females	19	8 (42.11)	11 (57.89)	
AFP (ng/mL)				0.327
<400	45	19 (42.22)	26 (47.78)	
>400	59	31 (52.54)	28 (47.56)	
Differentiation				<0.001*
High	23	5 (21.74)	18 (78.26)	
Middle	57	25 (43.86)	32 (56.16)	
Low	24	20 (83.33)	4 (16.67)	
Tumor size (d/cm)				0.010*
≤5	56	20 (35.71)	36 (64.29)	
>5	48	30 (62.50)	18 (37.50)	
Child				0.318
A	85	43 (50.59)	42 (49.41)	
B	19	7 (36.84)	12 (63.16)	

Note: *p* value was estimated by the chi-square test; *p < 0.05 was considered to be statistically significant; A, indicates well-compensated liver function; B, indicates significant functional compromise; PFDN6L, prefoldin subunit 6-like protein; AFP, Alpha-Fetoprotein.

### 2.11 Immunohistochemical (IHC) Staining

Human tumor tissues were fixed in paraformaldehyde, embedded in paraffin, sectioned, and subsequently subjected to dewaxing and rehydration, the tumor sections were blocked with BSA (Sigma, A7906, USA). Tissue sections were subsequently incubated overnight at 4°C with the CCDC30 polyclonal antibody (1:500; Thermo Fisher, PA5-57622, USA), followed by incubation with Biotin-SP–conjugated goat anti-human IgG (H + L) (1:1000; Proteintech, SA00004-6, Wuhan, China) for 20 min at 37°C after washing.DAB Detection Kit (Abcam, ab64238, UK) was used to develop the tissue samples and with hematoxylin solution at a concentration of 0.5% (w/v) to counterstain the nuclei. Staining assessment was conducted using a semi-quantitative scoring method that considered both the proportion of positively stained cells (0, none; 1, <1/100; 2, 1/100–1/10; 3, 1/10–1/3; 4, 1/3–2/3; 5, >2/3) and staining intensity (0, none; 1, weak; 2, moderate; 3, strong),yielding a total score of 0–8, with independent assessment by two blinded pathologists.

For xenograft tumor tissue analysis, paraffin-embedded sections from PLC/PRF/5 xenografts were subjected to IHC staining for PFDN6L, Ki67, CD133, and CD44 following the same protocol described above for patient-derived tissue samples. Primary antibodies used included anti-PFDN6L (Thermo Fisher, PA5-71297, 1:500), anti-Ki67 (Cell Signaling Technology, #9449, 1:400), anti-CD133 (Cell Signaling Technology, #5860, 1:3000), and anti-CD44 (Cell Signaling Technology, #37259, 1:4000).

### 2.12 Subcutaneous Xenograft Mouse Model

Male BALB/c nude mice (6 weeks old, 18–20 g) were obtained from Xuzhou Medical University (Xuzhou, China) and housed under specific pathogen-free conditions (22 ± 1°C, 55 ± 10% humidity, 12 h light/dark cycle) with free access to autoclaved food and water. All experimental protocols were approved by the Institutional Animal Care and Use Committee of Xuzhou Medical University (IACUC Approval No. KMMU2021713).

Mice were randomly assigned to experimental or control groups (n = 5 per group). Subcutaneous xenografts were generated by injecting 1 × 10^6^ Huh7 or PLC/PRF/5 cells in 100 μL PBS into the right flank. Tumor growth was monitored daily until palpable masses formed, with 10 mm maximum diameter established as the measurement threshold. Tumor size was measured every three days using digital calipers, and volumes were calculated as V = (L × W^2^)/2 (L: longest axis, W: perpendicular axis). Mice were humanely euthanized via CO_2_ > asphyxiation when tumors reached 1000 mm^3^ (PLC/PRF/5 group: 1500 mm^3^ as ethical endpoint), followed by necropsy and tumor excision for further analysis.

### 2.13 Statistical Analysis

Statistical analyses were conducted using SPSS software version 16.0 (IBM Corp., Armonk, NY, USA), and measurement data were presented as mean ± standard deviation (±s). The expression of PFDN6L on tissue microarray and clinical data was analyzed by chi-squared test. The survival rate was analyzed by Kaplan-Meier survival analysis and log-rank test. The independent prognostic factors were analyzed by Cox regression analysis. *t*-test was used to compare the two groups. The level of hypothesis test was determined by *p* < 0.05 was considered statistically significant. The statistical charts were drawn by GraphPad Prism 6 software (GraphPad Software, LLC, San Diego, CA, USA).

## 3 Results

### 3.1 The Expression of the PFDN6L Gene and Its Clinical Significance in Patients with HCC

**Figure 1:**
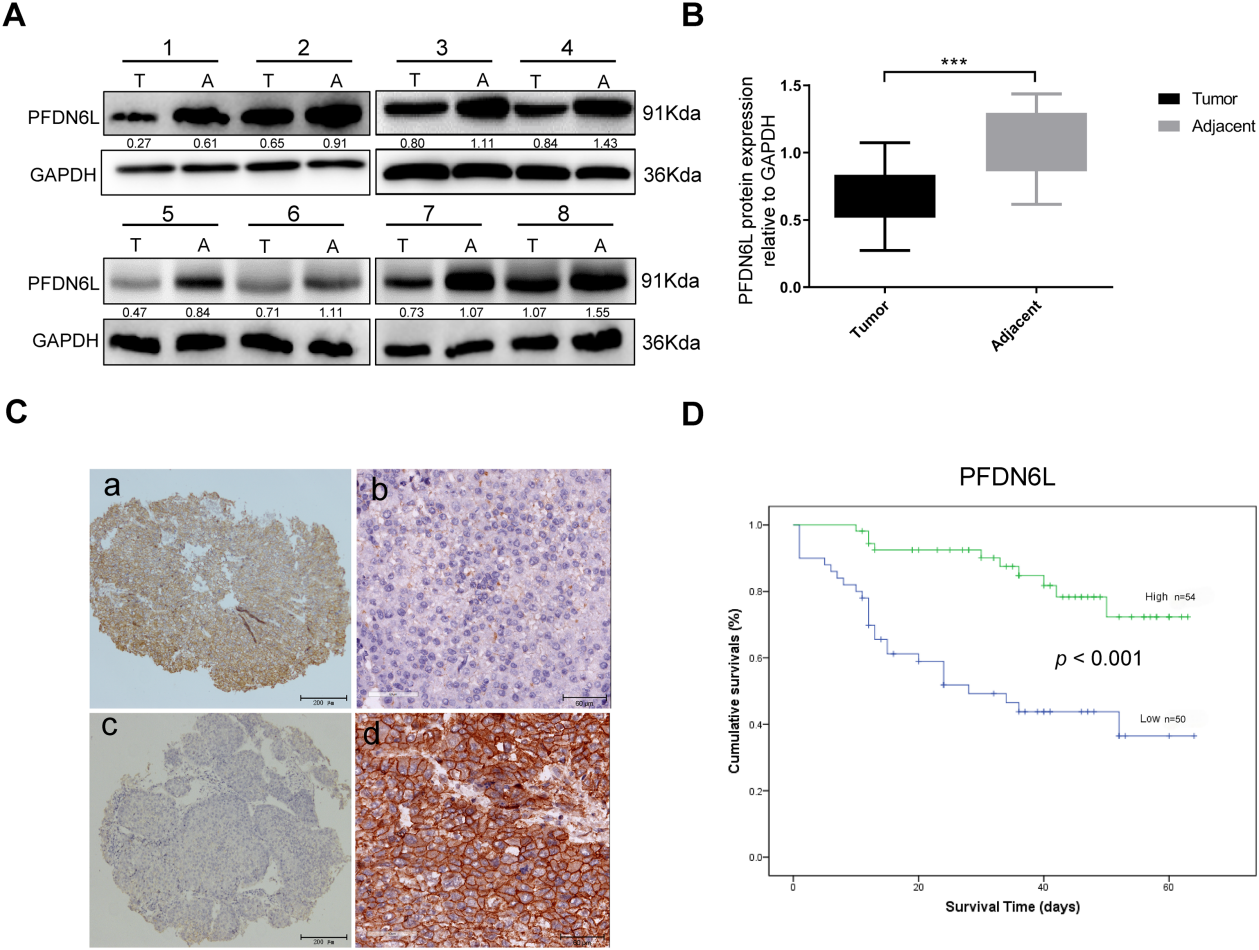

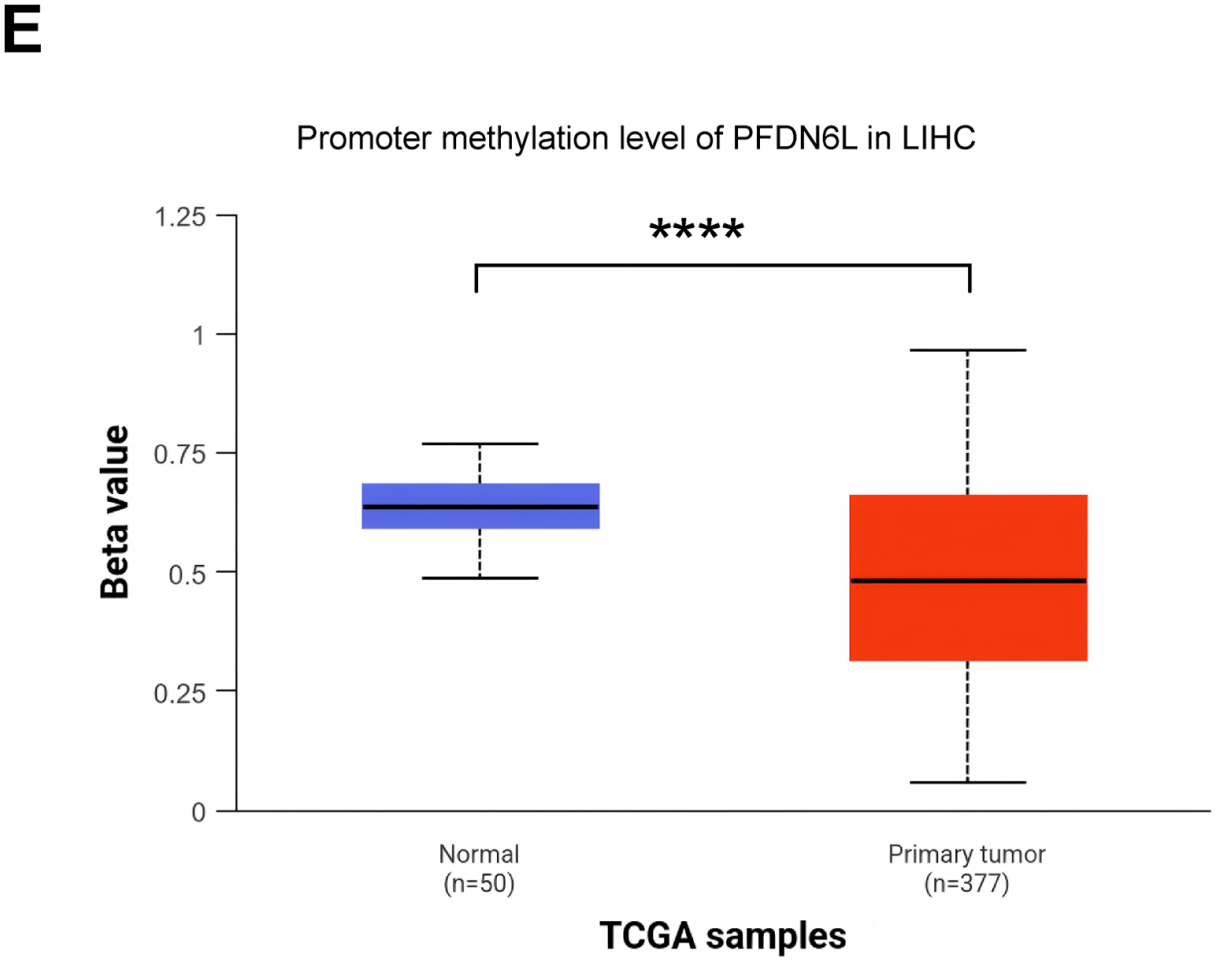
Immunohistochemistry and Western blot analyses revealed reduced PFDN6L expression in HCC tissues compared with adjacent non-tumorous tissues, and low tumor expression was associated with unfavorable prognosis. (**A**,**B**). The protein level of FFD6L was detected via western blot assays. GAPDH was used as a control. T represents cancer tissue, A represents para-cancerous tissue, and 1–8 denote the numbers of the tissues. (**C**) The levels of PFDN6L were detected by IHC assay on a human hepatocellular carcinoma tissue microarray. a and b indicate low expression of PFDN6L, while c and d indicate high expression of PFDN6L (microscopic magnification: a and c, ×40; b and d, ×400). (**D**) The association between PFDN6L expression and overall survival was assessed in a cohort of 104 HCC patients with follow-up data. (**E**) Expression patterns were compared across 377 HCC samples and 50 normal liver tissue samples from the TCGA databases. ***p < 0.001, ****p < 0.0001

We first determined the protein expression levels of PFDN6L in eight pairs of HCC samples and their adjacent tissues using western blot assay. Results indicated that PFDN6L protein levels were markedly reduced in HCC tissues compared with their paired adjacent non-tumorous tissues (Fig. 1A,B). (n = 8, Student’s *t*-test, *p* < 0.001). Subsequently, we utilized immunohistochemistry staining on a tissue microarray containing 104 HCC patient samples to explore the clinical significance of PFDN6L gene expression. The IHC results indicated that 50 out of 104 cases (48.07%) exhibited lower levels of PFDN6L expression. As depicted in Fig. 1C, the PFDN6L protein was predominantly localized in the cell membrane. In tumor tissues, lower PFDN6L expression was associated with poor tumor differentiation (*p* < 0.001) and larger tumor size (*p* = 0.01). However, no significant correlations were observed between PFDN6L expression and other clinical parameters, such as age, sex, serum alpha-fetoprotein (AFP) levels, or Child-Pugh classification (Table 1). We followed up all 104 patients to assess the impact of PFDN6L expression on the survival of HCC patients. Kaplan–Meier survival analysis showed that HCC patients with reduced PFDN6L expression exhibited significantly shorter overall survival than those with higher tumor expression levels (Fig. 1D) (*p* < 0.001). Furthermore, analysis of the TCGA dataset revealed that the protein level of PFDN6L was significantly lower in primary liver tumors compared to normal liver tissues (Fig. 1E) (*p* < 0.0001).

Univariate and multivariate Cox regression analyses were conducted to assess whether PFDN6L served as an independent prognostic factor in HCC. The univariate COX analysis indicated that the pathological stage, tumor size, serum AFP level, and PFDN6L expression were significant prognostic factors for the 5-year survival of HCC patients (Table 2). Moreover, in the multivariate COX regression analysis, as shown in Table 3, PFDN6L expression, tumor differentiation, and Child–Pugh classification were identified as independent predictors of 5-year survival in HCC patients. Collectively, the overexpression of PFDN6L may inhibit the progression of HCC.

**Table 2:** Univariate 5-year overall survival analysis in HCC patients

Variables	(n = 104 cases)	
	HR (95% CI)	p*
Age (≤60 years vs. > 60 years)	1.206 (0.631–2.303)	0.571
Sex (Males vs. Females)	1.033 (0.454–2.352)	0.938
Pathological stage (High, Middle vs. Low)	3.819 (2.116–6.893)	<0.001*
Tumor diameter (≤5 cm vs. >5 cm)	1.919 (1.000–3.681)	0.050*
AFP (>400 ng/mL vs. <400 ng/mL)	2.767 (1.305–5.868)	0.008*
Child	1.848 (0.893–3.821)	0.098
PFDN6L (Low vs. High)	0.241 (0.116–0.501)	<0.001*

Note: HR, hazard ratio; CI, confidence interval; *p* values are from the Log-rank test; *p < 0.05 was considered to be statistically significant; PFDN6L, prefoldin subunit 6-like protein; AFP, Alpha-Fetoprotein.

**Table 3:** Multivariate 5-year overall survival analysis in HCC patients

Variables	(n = 104 cases)	
	HR (95% CI)	p*
Age (≤60 years vs. >60 years)	1.233 (0.612–2.486)	0.558
Sex (Males vs. Females)	1.528 (0.589–3.966)	0.383
Pathological stage (High, Middle vs. Low)	2.929 (1.532–5.601)	0.001*
Tumor diameter (≤ 5 cm vs. >5 cm)	1.395 (0.665–2.928)	0.379
AFP (>400 ng/mL vs. <400 ng/mL)	1.945 (0.870–4.348)	0.105
Child	3.158 (1.390–7.174)	0.006*
PFDN6L (Low vs. High)	0.303 (0.135–0.682)	0.004*

Note. HR, hazard ratio; CI, confidence interval; *p* values are from the Log-rank test; *p < 0.05 was considered to be statistically significant; PFDN6L, prefoldin subunit 6-like protein; AFP, Alpha-Fetoprotein.

### 3.2 The Overexpression of the PFDN6L Gene Suppressed the Proliferation, Colony Formation of HCC Cell Lines, and Cell Cycle Progression

**Figure 2:**
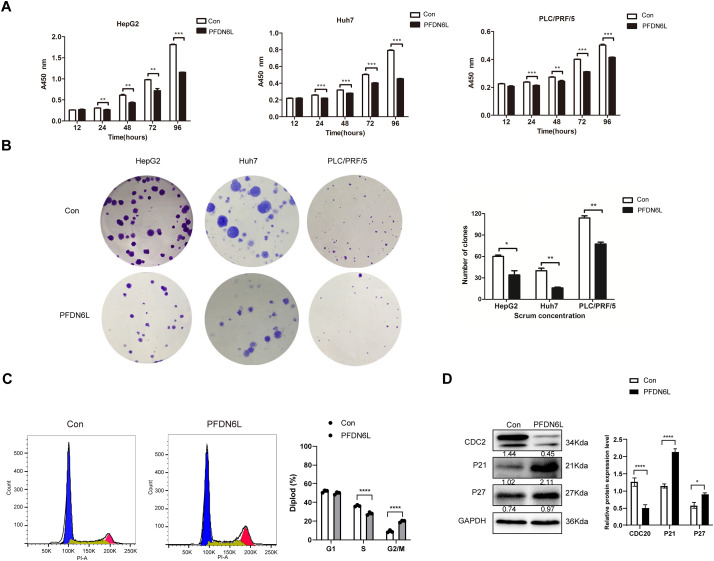
PFDN6L suppressed the proliferation and triggered cell cycle arrest in HCC cells. (**A**) The CCK-8 assay demonstrated that overexpression of PFDN6L inhibited the proliferation of HCC cells *in vitro*. (**B**) The number of cell clone formations was determined in HCC cells following the overexpression of PFDN6L. (**C**) Flow cytometry analysis revealed that overexpression of PFDN6L induced cell cycle arrest in PLC/PRF/5 cells. (**D**) Western blot analysis was carried out to examine the expression of proteins associated with the cell cycle, such as CDC2, p21, and p27. Data are presented as the mean ± SD of three independent experiments. *p < 0.05, **p < 0.01, ***p < 0.001, ****p < 0.0001

**Figure A1:**
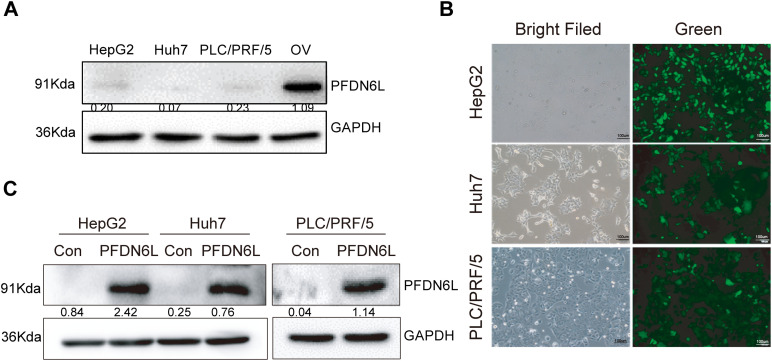
Construction of three stably HCC cell lines over-expressing PFDN6L. (**A**) Low expression of PFDN6L in HCC cell lines. OV (Oncolytic virus) represents transient over-expression of PFDN6L. (**B**) Fluorescent expression of HepG2, Huh7 and PLC/PRF/5 after stably transfection with pCDH-CMV-MCS-EF1-GFP+Puro. (**C**) Western-Blot analysis showed PFDN6L protein was over-expressed in HepG2, Huh7 and PLC/PRF/5

To elucidate the biological function of PFDN6L in LCCs, Western blot analysis was employed to detect the expression of PFDN6L in HepG2, Huh7, and PLC/PRF/5 cells. The findings revealed that the expression of PFDN6L was lower in these cells (Fig. A1A). Subsequently, we overexpressed PFDN6L in three types of HCC cell lines using lentiviral vectors pCDH-CMV-MCS-EF1-GFP+Puro. The infection efficiency was monitored via a fluorescence microscope (Fig. A1B). As presented in Fig. A1C, transfection with the PFDN6L gene significantly upregulated the expression of the PFDN6L protein in HCC cells. To assess the impact of PFDN6L overexpression on cell proliferation, we utilized CCK8 and colony-formation assays. As shown in Fig. 2A, the OD values of HepG2 cells overexpressing PFDN6L at 24, 48, 72 and 96 h were 0.14, 0.29, 0.28 and 0.36 times lower than those of the control group, respectively. Similar to HepG2 cells, the CCK8 OD values of Huh7 and PLC/PRF/5 cells overexpressing PFDN6L were also significantly lower than those of the control group (Table A1).

**Figure A2:**
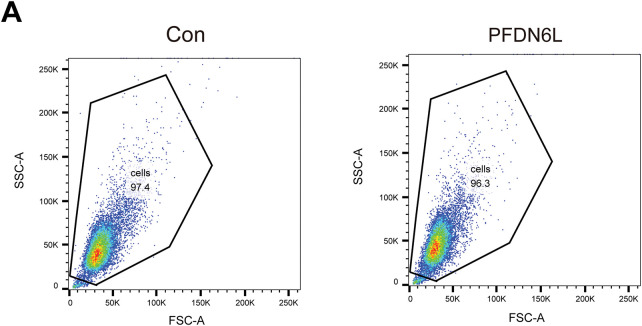

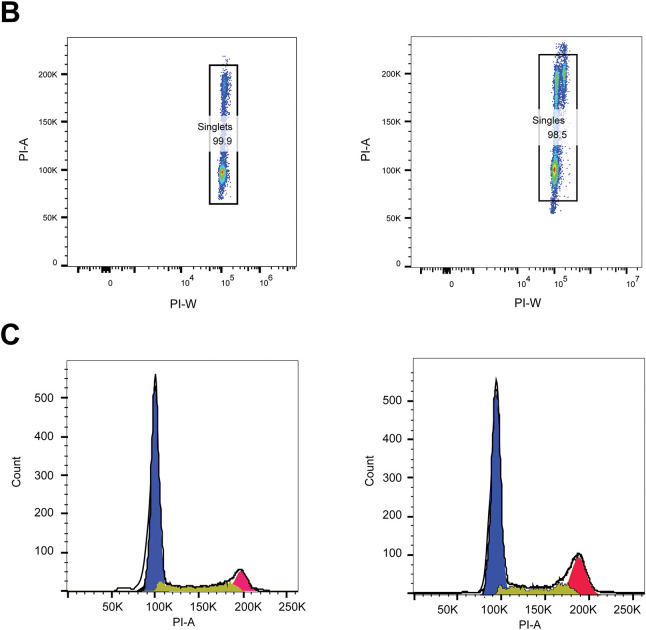
The representative flow cytometry gating strategies and validation of Fig. 2C. (**A**) FSC-A vs. SSC-A (debris exclusion) → Live PLC/PRF/5 cells. (**B**) PI-W vs. PI-A, Name this gate: Singlets. (**C**) Select DNA Stain Channel for Cell Cycle Analysis, Histogram of DNA dye PI-A applied to Singlets

We also demonstrated that the number and size of monoclonal colonies formed in PFDN6L-overexpressed HepG2, Huh7, and PLC/PRF/5 cells were significantly lower than those in the control group. Statistical analysis showed that the number of colonies in PFDN6L-overexpressed HepG2, Huh7, and PLC/PRF/5 cells was 0.48, 0.55, and 0.29 times lower than that of the controls, respectively. Moreover, the colony size in the PFDN6L group was smaller than that in the control group (Fig. 2B). These results indicated that overexpression of PFDN6L significantly inhibited the proliferation and colony formation ability of HCC cells. We further verified the effect of the PFDL6 gene on the cell cycle using annexin5/PI staining. The cell cycle profiles for control PLC/PRF/5 cells and those overexpressing PFDN6L were as follows: G1 phase: 51.83 ± 1.09% vs. 49.77 ± 1.14%; S phase: 36.30 ± 0.75% vs. 27.99 ± 1.25%; G2/M phase: 8.98 ± 1.03% vs. 19.73 ± 0.93% (*p* < 0.0001) (Figs. 2C and A2 and Table A2). This finding indicated that PFDN6L overexpression significantly increased the proportion of cells in the G2/M phase, suggesting induction of G2/M arrest. In addition, we employed Western blotting assays to explore the underlying mechanism. The results revealed that PFDN6L downregulated the expression of CDC2, a cyclin-dependent protein kinase, while upregulating the expression of cyclin-dependent kinase inhibitors p21 and p27 (Fig. 2D). These results further confirmed that overexpression of PFDN6L induced cell cycle arrest.

### 3.3 PFDN6L Inhibited the Stem Cell Characteristics of HCC

**Figure 3:**
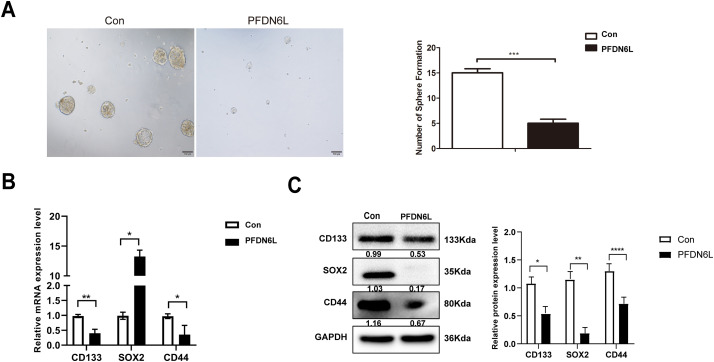
PFDN6L suppressed the stem cell characteristics of HCC. (**A**) The impact of PFDN6L overexpression on the *in vitro* sphere formation ability of HCC stem cells was analyzed via an *in vitro* sphere formation assay. (**B**) qRT-PCR results showed the transcript levels of PFDN6L, CD133, CD44, and Notch1 in the control group and the PFDN6L overexpression group. (**C**) The protein expression levels of CD133, CD44, and SOX2 were measured by Western blot. Data are presented as the mean ± SD of three independent experiments. *p < 0.05, **p < 0.01, ***p < 0.001, ****p < 0.0001

HCC cells display stronger stem cell-like characteristics, including migration, spheroidization, and tumorigenicity. To investigate the effect of PFDN6L on the self-renewal potential of liver cancer stem cells, an *in vitro* sphere-forming assay was performed. Previous studies have reported that CD133 serves as a marker for HCC stem cells [17]. Therefore, CD133-positive (CD133^+^) HCC stem cells were isolated from PLC/PRF/5 cells overexpressing PFDN6L and their control counterparts using magnetic bead sorting technology. Subsequently, CD133^+^ PLC/PRF/5 cells were collected from the PFDN6L overexpression group and the control group, and the effect of PFDN6L on the sphere formation ability of these cells was detected via the Sphere forming assay. As depicted in Fig. 3A, the number of spheres formed by cells overexpressing PFDN6L was significantly fewer than that of the control group, and the volume of the formed spheres was also significantly smaller. The number of spheres formed by cells overexpressing PFDN6L (n = 5 ± 0.82) was significantly reduced compared with the control group (n = 15 ± 0.82). These findings suggest that overexpression of PFDN6L inhibits the *in vitro* sphere formation ability of HCC stem cells.

CD44, CD133, notch1, and SOX2 are recognized as marker genes of HCC stem cells. To ultimately clarify the stem cell effect of PFDN6L on HCC stem cells, quantitative polymerase chain reaction (qRT-PCR) and Western blot analysis were employed to detect the regulatory impact of PFDN6L on stem cell-related genes. PFDN6L overexpression resulted in decreased mRNA levels of CD133, CD44, and Notch1 genes (Fig. 3B). Additionally, overexpression of PFDN6L decreased the protein expression levels of CD133, SOX2, and CD44 genes (Fig. 3C). Collectively, these findings indicate that PFDN6L inhibits the stem cell characteristics of HCC.

### 3.4 PFDN6L Suppresses the Subcutaneous Xenograft Tumorigenesis and Tumor Growth of HCC

**Figure 4:**
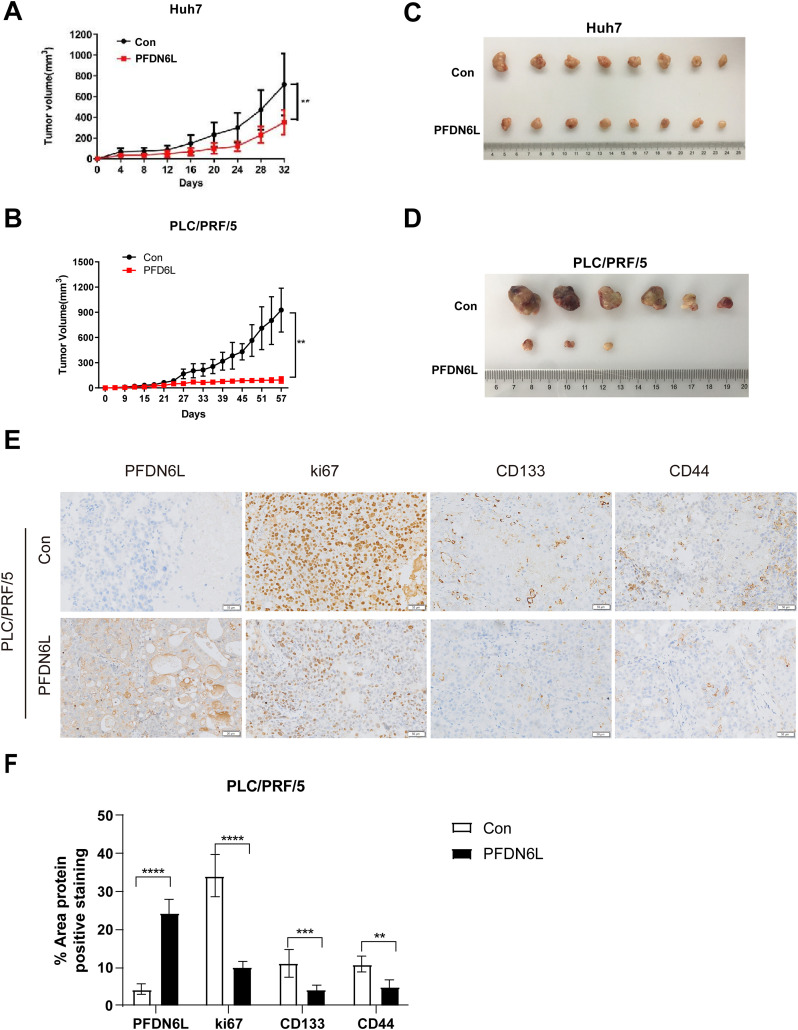
Overexpression of PFDN6L inhibited the growth of subcutaneous tumors. (**A,B**) The tumor growth curve was recorded and quantified. (**C,D**) Xenograft tumor images of Huh7 and PLC/PRF/5 cells from the control group and the PFDN6L group. (**E,F**) Immunohistochemical staining images of PFDN6L, Ki67, CD133 and CD44 in xenograft tumors of the control group of PLC/PRF/5 cells and the PFDN6L group (magnification ×400). **p < 0.01, ***p < 0.001, ****p < 0.0001

To evaluate the *in vivo* effect of PFDN6L, subcutaneous xenograft tumors were generated in nude mice. The mice were allocated into an experimental group and a control group. Overexpression PFDN6L Huh7 cells (1 × 10^6^ cells), overexpression PFDN6L PLC/PRF/5 cells (1 × 10^6^ cells), or their respective control groups were injected into both flanks of nude mice. As depicted in Fig. 4A,B and presented in Table 4, in the Huh7 cells control group, all tumors formed on the 4th day following subcutaneous injection. In the overexpression PFDN6L group, all tumors formed on the 6th day. There was no significant difference in tumorigenicity. However, overexpression of PFDN6L could inhibit the *in vivo* proliferation of tumor cells (*p* < 0.01). In the Control groups of PLC/PRF/5, tumorigenesis occurred on the 18th day after cell inoculation, respectively. In contrast, in the overexpression PFDN6L group, only 50% tumorigenesis was observed on the 56th day after cell inoculation (Table 5). The tumorigenic ability was significantly diminished, and overexpression of PFDN6L significantly inhibited the *in vivo* proliferation ability of tumor cells (*p* < 0.01, Fig. 4C,D). Furthermore, to investigate the underlying mechanism through which PFDN6L suppresses tumor growth *in vivo*, tumor tissue was harvested from PLC/PRF/5 xenograft mice, and immunohistochemical staining was carried out. The expressions of PFDN6L, Ki67, CD133, and CD44 were detected. Fig. 4E illustrates that PFDN6L expression was elevated in the overexpression group compared with the control group. Moreover, Ki67 expression was markedly reduced in the PFDN6L overexpression group compared with the control group.Additionally, results showed that CD133 and CD44 expression levels were reduced in the PFDN6L overexpression group relative to the control group (Fig. 4E,F). These findings suggest that the overexpression of PFDN6L can inhibit the tumorigenicity and growth of HCC cells *in vivo*.

**Table 4:** The effect of PFDN6L in Huh7 cells on tumorigenicity

Number of injected cells		No. of mice with tumor formation/Total No. of mice with cell injection					
		1 d	2 d	3d	4d	5 d	6 d
1 × 10^6^	Con	0/8	4/8	6/8	8/8	8/8	8/8
	PFDN6L	0/8	2/8	5/8	6/8	6/8	8/8

**Table 5:** The effect of PFDN6L in PLC/PRF/5 cells on tumorigenicity

Number of injected cells		No. of Mice with tumor formation/Total No. of mice with cell injection									
		6d	9d	12d	15d	18d	21d	24d	27d	…	56d
1 × 10^6^	Con	0/6	1/6	1/6	4/6	6/6	6/6	6/6	6/6	…	6/6
	PFDN6L	0/6	0/6	0/6	0/6	1/6	2/6	2/6	3/6	…	3/6

### 3.5 PFDN6L Overexpression Inhibits HCC Cell Proliferation through Suppression of the AKT and ERK1/2 Signaling Pathways

**Figure 5:**
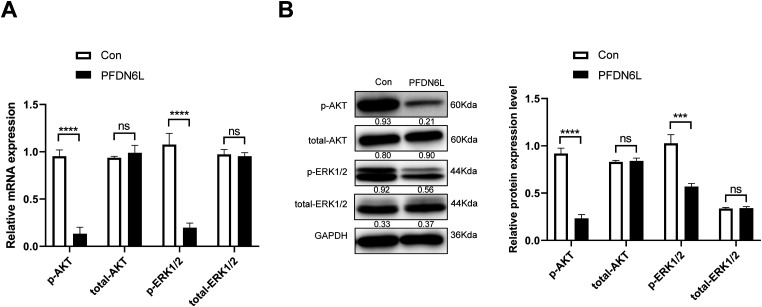
Western blot analysis showed that PFDN6L reduced AKT and ERK1/2 phosphorylation. (**A**) mRNA levels of p-AKT, total-AKT, p-ERK1/2, and total-ERK1/2 were measured by qRT-PCR. (**B**) Protein levels of p-AKT, total-AKT, p-ERK1/2, and total-ERK1/2 were assessed via western blotting. Data represent mean ± SD from three independent experiments. ns, not significant; ***p < 0.001, ****p < 0.0001

To further investigate the mechanism of PFDN6L regulation on HCC cells’ proliferation, we examined the expression of genes associated with the pathway. Compared with the control group, PFDN6L overexpression reduced both mRNA and protein levels of phosphorylated AKT and ERK1/2, suggesting that it suppresses HCC cell proliferation by inhibiting the AKT and ERK1/2 signaling pathways (Fig. 5A,B).

## 4 Discussion

PFDN6L is a recently discovered gene that may function as a novel cytoskeletal element associated with the cytoskeleton [20]. This study focused on elucidating the biological role and molecular mechanisms of PFDN6L in the regulation of cancer stem cells in HCC. We demonstrated that PFDN6L functions as a tumor suppressor, and its expression level correlates with key pathological features of HCC. These observations, in conjunction with survival data and multivariate analysis, suggest the potential prognostic value of PFDN6L in HCC. The establishment of PFDN6L-overexpressing cell lines allowed us to further elucidate its biological role. Importantly, the findings indicate that PFDN6L overexpression exerts inhibitory effects on HCC cell behavior. These findings underscore the significance of PFDN6L in HCC progression and its promise as a potential therapeutic target.

Cell cycle arrest mediates proliferation inhibition. The cyclin/CDK complex drives cell cycle progression, whereas CKIs suppress this process [22]. Flow cytometry and Western blot analyses demonstrated that PFDN6L overexpression induced G2/M phase arrest, concomitant with CDC2 downregulation and p21/p27 upregulation. As a cyclin-dependent kinase, CDC2 facilitates G2/M transition and serves as a critical checkpoint regulator [23]. The CKIs p21 and p27 negatively regulate cell cycle progression [24]. Thus, PFDN6L inhibits proliferation by suppressing CDC2 while enhancing p21/p27, thereby blocking G2/M transition.

Findings from the *in vitro* sphere formation assay, aimed at evaluating the self-renewal capacity of HCC stem cells, indicated that PFDN6L inhibited the proliferation of HCC cells. Additionally, *in vivo* studies revealed that overexpression of PFDN6L suppressed the tumorigenicity and tumor growth of HCC cells. Taken together, these results indicate that PFDN6L overexpression suppresses tumor cell proliferation in both *in vitro* and *in vivo* settings. Subsequently, we explored the mechanism by which PFDN6L regulates the proliferation of hepatocellular carcinoma. The PI3K/AKT signaling pathway is pivotal in controlling cell proliferation and differentiation. Activation of phosphorylated AKT can initiate the PI3K/AKT signaling pathway, thereby promoting the development of malignant tumors [25]. Reports indicate that the PI3K/Akt/mTOR pathway promotes liver cancer stem cell formation and drives the malignant progression of liver cancer [26].

The ERK1/2 signaling pathway is also essential for controlling the proliferation, migration, and invasion of malignant cells [27]. The RAS/RAF/MEK/ERK signaling pathway controls the progression of various malignant tumor tissues, including liver cancer [28]. Studies have indicated that inhibiting the phosphorylation of ERK1/2 and blocking the RAS/RAF/ERK signaling pathway can hinder tumor progression [29]. Our research demonstrated that overexpressing PFDN6L downregulated the expression of phosphorylated AKT and ERK1/2 and significantly inhibited the activation of AKT and ERK1/2. Thus, PFDN6L may exert its tumor-inhibitory effect by negatively regulating the PI3K/AKT and ERK1/2 signaling pathways in hepatocellular carcinoma. It has been reported that RAS is regarded as the main upstream regulator of the AKT and ERK1/2 signal transduction mechanisms [30]. Notably, recent studies demonstrate that dual AKT/ERK blockade synergistically disrupts LCSC self-renewal and overcomes sorafenib resistance [31]. Further investigation into PFDN6L’s role in RAS palmitoylation—a key modulator of membrane localization and oncogenic signaling [32]—could reveal novel therapeutic targets. Therefore, we hypothesize that overexpressing PFDN6L may inhibit the activation of RAS, thereby leading to the inactivation of AKT and ERK1/2. This aspect remains unresolved and requires further investigation for confirmation.

CD133 and CD44 serve as surface markers of liver cancer stem cells. Among them, CD133 is regarded as a highly important surface marker of tumor stem cells and is implicated in the malignant progression of liver cancer [31]. qRT-PCR and Western blot findings demonstrated that the overexpression of PFDN6L leegulation of the mRNA expression levels of CD133, CD44, and Notch1, as d to a downrwell as a reduction in the protein expression levels of CD133, SOX2, and CD44. Prior studies have demonstrated that suppressing CD133 expression increases the sensitivity of liver cancer cells to chemotherapy and radiotherapy while attenuating their malignant progression [32]. Notch1 is highly expressed in liver cancer tissue [33]. Activation of the Notch1 signaling pathway enhances the stemness of liver cancer stem cells and drives liver cancer progression [34]. SOX2, a key transcription factor in preserving stem cell traits, plays a role in the initiation and malignant progression of multiple tumor types [35]. Research has shown that SOX2 expression is strongly linked to liver cancer metastasis and patient prognosis. SOX2 activates cell invasion and epithelial-mesenchymal transition (EMT) by regulating the expression of Slug in liver cancer cells [36]. Additionally, AKT can directly interact with SOX2 and enhance its own stability through phosphorylation at the Thr118 site, thereby increasing the transcriptional activity of SOX2 [37].

We therefore propose that PFDN6L overexpression may suppress the self-renewal and tumorigenic capacity of hepatocellular carcinoma (HCC) stem cells by modulating stem cell–associated genes, thereby reducing HCC metastasis and recurrence. In our study, the protein level of SOX2 was significantly downregulated after the overexpression of PFDN6L. However, the mechanism by which PFDN6L regulates SOX2 remains unclear and future studies should assess whether PFDN6L disrupts chaperone networks governing SOX2 stability or modulates metabolic dependencies (e.g., fatty acid oxidation) in LCSCs, as these pathways are increasingly linked to therapy resistance.

Although our results emphasize the tumor-suppressive function of PFDN6L in HCC and its role in regulating liver cancer stem cells, several limitations must be noted. Foremost, the relatively small number of patient tissue samples used for correlation and survival analyses may restrict the broader applicability of these findings. Second, although *in vitro* and *in vivo* assays supported the role of PFDN6L in tumor suppression, the direct upstream regulators and complete downstream signaling network of PFDN6L remain to be elucidated. Third, the potential impact of PFDN6L on therapy resistance or drug sensitivity in HCC was not evaluated in this study. Additional studies involving larger patient cohorts and in-depth mechanistic analyses are needed to confirm and expand upon our current findings.

## 5 Conclusion

PFDN6L was lowly expressed in HCC and this low expression was associated with tumor size and tumor differentiation. Furthermore, over-experssion of PFDN6L suppresses HCC proliferation *in vitro*, tumorigenesis and tumor growth *in vivo*. And it inhibited to the self-renewal of HCC stem cells. PFDN6L may serve as a promising therapeutic target for treating human HCC.

## Data Availability

Data used in this study is available with the communicating authors upon reasonable request for non-commercial purposes.

## Appendix A

**Table A1:** The OD Value was detected by CCK-8 assay of each group

Cells	Groups	A 450 nm ($\bar{\text{x}}$ ± *s*, n = 3)				
		12 h	24 h	48 h	72 h	96 h
HepG2	Con	0.26 ± 0.002	0.31 ± 0.002	0.61 ± 0.021	0.98 ± 0.007	1.81 ± 0.009
	PFDN6L	0.27 ± 0.009	0.27 ± 0.009	0.43 ± 0.013	0.72 ± 0.051	1.16 ± 0.003
Huh7	Con	0.22 ± 0.003	0.26 ± 0.003	0.32 ± 0.002	0.50 ± 0.005	0.79 ± 0005
	PFDN6L	0.22 ± 0.003	0.22 ± 0.001	0.28 ± 0.001	0.40 ± 0.003	0.45 ± 0.003
PLC/PRF/5	Con	0.23 ± 0.002	0.24 ± 0.001	0.27 ± 0.002	0.40 ± 0.000	0.50 ± 0.007
	PFDN6L	0.21 ± 0.003	0.21 ± 0.003	0.24 ± 0.006	0.31 ± 0.002	0.41 ± 0.002

**Table A2:** The Distribution of the cell cycle in each group

Cells	Groups	Cell cycle distribution (%, $\bar{\text{x}}$ ± *s*, n = 3)		
		G1	S	G2/M
PLC/PRF/5	Con	51.83 ± 1.09	36.30 ± 0.75	8.98 ± 1.03
	PFDN6L	49.77 ± 1.14	27.99 ± 1.25	19.73 ± 0.93
